# Reducing patient delay in acute coronary syndrome: Randomized controlled trial testing effect of behaviour change intervention on intentions to seek help

**DOI:** 10.1111/bjhp.12619

**Published:** 2022-08-08

**Authors:** Barbara Farquharson, Marie Johnston, Brian Williams, Karen Smith, Stephan Dombrowski, Claire Jones, Shaun Treweek, Nadine Dougall, Mark Grindle, Jan Savinc, Purva Abyhankar

**Affiliations:** ^1^ University of Stirling Stirling UK; ^2^ University of Aberdeen Aberdeen UK; ^3^ Edinburgh Napier University Edinburgh UK; ^4^ University of Dundee Dundee UK; ^5^ University of New Brunswick Fredericton Canada; ^6^ University of Highlands and Islands Inverness UK

**Keywords:** acute coronary syndrome, BCT, behaviour, behaviour change, cardiac, delay, intervention, patient delay

## Abstract

**Objectives:**

The aim of this study was to evaluate the efficacy of a behaviour change intervention to reduce patient delay with symptoms of acute coronary syndrome.

**Design:**

A 3‐arm web‐based, parallel randomized controlled trial.

**Methods:**

The intervention comprised 12 behaviour change techniques (BCTs) embedded in a text‐only or text+visual narrative (the techniques were systematically identified through systematic review and a consensus exercise). Between February and November 2017, *n* = 145 people who had recently experienced acute coronary syndrome were randomly allocated to intervention (‘text+visual’ or ‘text‐only’) or control. Intentions to phone an ambulance immediately for acute coronary syndrome symptoms were assessed before and after the intervention using symptom scenarios, and the change in intention was compared across the three groups.

**Results:**

Significant increases in intention to phone an ambulance immediately for ACS symptoms were seen following the ‘text+visual’ intervention but not following ‘text‐only’ or control. However, the study was underpowered to detect any significant changes in intention between the 3 groups. There were no unintended effects on intentions for non‐urgent symptoms.

**Conclusions:**

A ‘text+visual’ BCT‐based intervention may significantly increase intention to phone an ambulance with symptoms of ACS. Further testing of the effect of the intervention on actual behaviour is required.

**Statement of contribution**

*What is already known on this subject?*

- Patient delay with symptoms of acute coronary syndrome is common and associated with increased mortality.
- Trials of interventions designed to reduce patient delay have had mixed results.
- It is not clear what the critical ‘active ingredients’ of an intervention to reduce patient delay are—behaviour change techniques might be useful.

*What does this study add?*

- The study confirms a behaviour change intervention designed to reduce patient delay is acceptable and engaging to people with previous Acute Coronary Syndrome.
- The text and visual behaviour change intervention (animation) can significantly increase intentions to phone an ambulance with symptoms of acute coronary syndrome without increasing intentions for non‐serious symptoms.
- This test of the intervention was particularly robust, requiring people to respond to a range of undifferentiated symptom scenarios, and as such these results provide more compelling evidence for further evaluation than studies which only demonstrate improved patient knowledge about symptoms.

## INTRODUCTION

It has long been recognized that early access to advanced life support and treatment is critical to reducing mortality and morbidity in acute coronary syndrome (ACS) (Ho et al., 1989). The well‐documented benefits of treatment are time‐dependent, and maximum benefit is only achieved when treatment is given promptly (ideally within 2 hours of symptom onset) (Boersma, 2006; Collet et al., 2020; Ibanez et al., 2017). Contemporary health care systems invest a great deal to ensure patients with suspected ACS can be assessed and treated quickly: Emergency telephone services, rapid response by paramedic, transfer to hospital by ambulance and prompt initiation of treatments such as percutaneous coronary intervention or thrombolysis (Ibanez et al., 2017; O'Connor et al., 2010) have successfully reduced delays once people present symptoms to health care providers. However, the main component of delay actually occurs before the patient enters the health care system (G.I.p.l.S.d.S.n.I. (GISSI), 1995; Nilsson et al., 2016). This interval, between the onset of symptoms and seeking medical help, is known as ‘patient decision time’ or ‘patient delay’. Average patient delay times range from 1.5 to 7 hrs (Dracup et al., 2003), leaving many patients outside the optimal window for treatment before they even reach hospital (Eagle et al., 2002; McNair et al., 2019). Recently, the COVID‐19 pandemic may have further exacerbated the problem (Toner et al., 2020). Most deaths due to ACS occur pre‐hospital (Chambless et al., 1997; Grey et al., 2017; Vervueren et al., 2012), deaths which cannot be avoided by in‐hospital strategies. Patient delay is potentially modifiable and if reductions in pre‐hospital time can be achieved, this has the potential to significantly reduce the number of deaths from ACS (Grey et al., 2017; Norris, 2005).

## BACKGROUND

Considerable success has been achieved in reducing service components of treatment delay in ACS (e.g. ambulance response and ‘door‐to‐needle’ times), but patient delay has proved more challenging to reduce (Ibanez et al., 2017; Saczynski et al., 2008; Schiele et al., 2010). Interventions aimed at reducing patient delay times for ACS and other time‐critical conditions have been evaluated, but results are mixed (Farquharson et al., 2019). Approximately half of interventions demonstrate a significant effect on delay, and this proportion is similar across study designs, mode of delivery, populations and clinical contexts (Farquharson et al., 2019). The content of one of the most successful recent interventions (Mooney et al., 2014) is strikingly similar to that of a previous trial which reported no significant reduction in delay (Dracup et al., 2009). The critical ‘active ingredients’ of an intervention to reduce patient delay therefore remain unclear.

The publication of the BCT Taxonomy (BCTTv1) (Michie et al., 2013) provides an opportunity to systematically and explicitly specify the content of behaviour change interventions and thus to better identify critical ‘active ingredients’. The taxonomy also allows us to go beyond establishing only whether complex behaviour change interventions ‘work’ and move towards a better understanding of ‘how’ they work (Sumner et al., 2018). The theory of planned behaviour (TPB) (Ajzen, 1991a; McEachan et al., 2011), social cognitive theory (Bandura, 1986) and Leventhal's Commonsense Model of Self‐Regulation (Farquharson et al., 2012; Leventhal et al., 1980; Walsh et al., 2004) were considered likely to be helpful in explaining ‘how’ delay behaviour was modified, and so constructs derived from these models were measured as secondary outcomes.

A systematic review of the BCT content of previous interventions aimed at reducing delay with symptoms was intended to identify effective BCTs (optimal content) (see Farquharson et al., 2014 for details of methods). The level of BCT content varied markedly in interventions, ranging from 0 to 10 (average: 2 techniques per intervention). Unfortunately, the review was not helpful in identifying which BCTs were associated with effective interventions: Individual BCTs were as frequently found in ineffective interventions as they were in effective ones. However, a greater number of BCTs might be important in relation to reducing patient delay: three (Luiz et al., 2001; Rowley et al., 1982; Wolters et al., 2015) of the four studies which included 2 or more BCTs reported a significant reduction in patient delay (Farquharson et al., 2019).

In parallel with the SR, we also conducted a pre‐specified (see protocol for detailed methods (Farquharson et al., 2017)) consensus study (modified Delphi (Hsu & Sandford, 2007)) with *n* = 12 behaviour change experts. This transparent process identified twelve BCTs that experts judged were necessary for inclusion in an intervention to reduce patient delay (see Box 1). The interventions were systematically developed to include the 12 identified BCTs.

Box 1 1.2 Problem‐solving

1.4 Action planning

3.2 Social support (practical)

3.3 Social support (emotional)

4.1 Instruction on how to perform the behaviour

5.1 Information about health consequences

5.2 Salience of health consequences

7.1 Prompts/cues

9.1 Credible source

9.2 Pros and cons

9.3 Comparative imagining of future outcomes

15.2 Mental rehearsal of successful performance

We also sought to establish whether a visual mode of delivering the BCT content might be more effective than non‐visual. A visual mode of delivery offers a number of potential advantages such as being more emotionally evocative (Paivio et al., 1994), memorable (Leventhal et al., 1984) and accessible (e.g. to people with low literacy levels) (Steele et al., 2007), but evidence of benefit over non‐visual for otherwise identical content was absent. We therefore created and tested two versions of a BCT‐based intervention: a ‘text+visual’ version and a ‘text‐only’ version.

The ultimate goal is to reduce patient delay behaviour. However, in line with published guidance for evaluating complex interventions (Craig et al., 2008; Eldridge et al., 2016), we elected to first test the effectiveness of the intervention in modifying ‘intentions’, a crucial first step in changing behaviour (Orbell & Sheeran, 1998). The intervention was trialled in people with a history of previous ACS events as this is a group at the highest risk of ACS and thus a priority group for intervention (Anderson et al., 2013; Ibanez et al., 2017). We were cognisant that an intervention which encourages people to seek help quickly with particular symptoms could have unintended effects on response to other symptoms, perhaps unnecessarily burdening health services. Therefore, the trial also tested the effect of the intervention on non‐critical symptoms (toe pain and discomfort passing urine).

## AIMS

1. To test the effectiveness of the theory‐based interventions (‘text+visual’ and ‘text‐only’ BCT‐based interventions) against usual care in changing patients' intentions to phone ambulance immediately with symptoms of ACS ≥15 minutes duration.
2. To determine the more effective mode of delivery by comparing the ‘text+visual’ BCT‐based intervention and ‘text‐only’ BCT‐based intervention, with the usual care group.
3. To investigate any unintended consequences of the intervention on intentions to phone an ambulance for non‐life‐threatening symptoms.

## METHODS

### Design

An intervention modelling experiment (IME) conducted as a parallel 3‐arm (text+visual, text‐only, control) randomized controlled trial. The study was web‐based and conducted via a bespoke web‐based interface. The protocol, with methods described in full detail, was published ahead of the trial, and thus, some of the methods reported below are unavoidably duplicated (Farquharson et al., 2017). In brief, participants indicated their intentions for a range of ACS and non‐ACS symptom scenarios before receiving the intervention as per allocation, after which they again responded their intentions to another set of symptom scenarios.

### Setting and participants

Potential participants were adults (aged >18 years) who had experienced ACS within the previous 6 months and were identified via 2 routes.

1. From three NHS board areas in Scotland.
2. From the Scottish Health Research Register (SHARE) (http://www.registerforshare.org), a register of people interested in participating in health research.

#### Inclusion criteria

Adults, aged >18 years, who have experienced ACS within the previous 6 months.

#### Exclusion criteria

Anyone still admitted to hospital.

People who had experienced ACS within the previous 2 weeks.

### Sample size

We originally intended to recruit a sample size of 177 participants which was calculated to provide a 90% power of detecting an effect size of 0.66 at a significance level of 0.025.

### Data collection

#### Recruitment via NHS site

People admitted to NHS sites with confirmed ACS were identified from hospital records by the local cardiac rehabilitation team. Initially, the cardiac rehabilitation team provided an invitation letter and participant information sheet to all eligible patients and those who wished to participate were asked to contact the research team. However, this resulted in a slower than expected response, and so, after 7 participants had been recruited, procedures were revised (with ethical approval): (1) Potential participants were given the opportunity to opt out. Those who did not opt‐out were contacted by telephone and given the opportunity to discuss participation with a researcher. (2) Eligible patients attending cardiac rehabilitation classes were offered the opportunity to take part before or after their cardiac rehabilitation class. (3) Compensation (£10 voucher) was offered to a randomly selected 50% of potential participants to evaluate whether it had any impact on recruitment.

#### Recruitment via SHARE

The SHARE office identified and approached SHARE registrants who met the study eligibility criteria with a brief description of the proposed study. Upon receiving an indication of interest in the study from the potential volunteers, a letter of invitation along with the participant information sheet was provided to the potential volunteers. A reminder letter was sent 2 weeks after the original letter to those who had not responded.

### Consent procedure

The study information sent to potential participants contained a unique participant code and details of how to access and take part in the web‐based study. Those who wished to participate visited the study web link to provide confirmation of consent and take part in the study. Those who did not wish to participate simply did not access the web link and were not contacted again.

### Randomisation

Following informed consent, baseline demographic and study data were collected before participants were randomly allocated to one of the three study groups. Allocation was concealed from participants until they received the intervention. The randomisation was managed by the web‐based study software. To ensure balance across the three groups, a random permuted blocks approach was adopted with seed value recorded. Participants were randomized to the next available block through the study website after providing positive consent on the consent screen.

### Intervention

#### Control condition (usual care)

Participants in the control group received information that was currently used routinely in the NHS site to inform patients with ACS what to do whether they experienced symptoms after discharge. All the usual care information explained the symptoms of angina and heart attack and advised what to do in the event of experiencing these symptoms. The information was presented in written text format on screen. BCT coding of the usual care information identified that each board area's provided information with one BCT: *4.1 Instruction on how to perform the behaviour*.

#### Usual care plus ‘text+visual’ BCT‐based intervention (intervention group 1)

**FIGURE 1:**
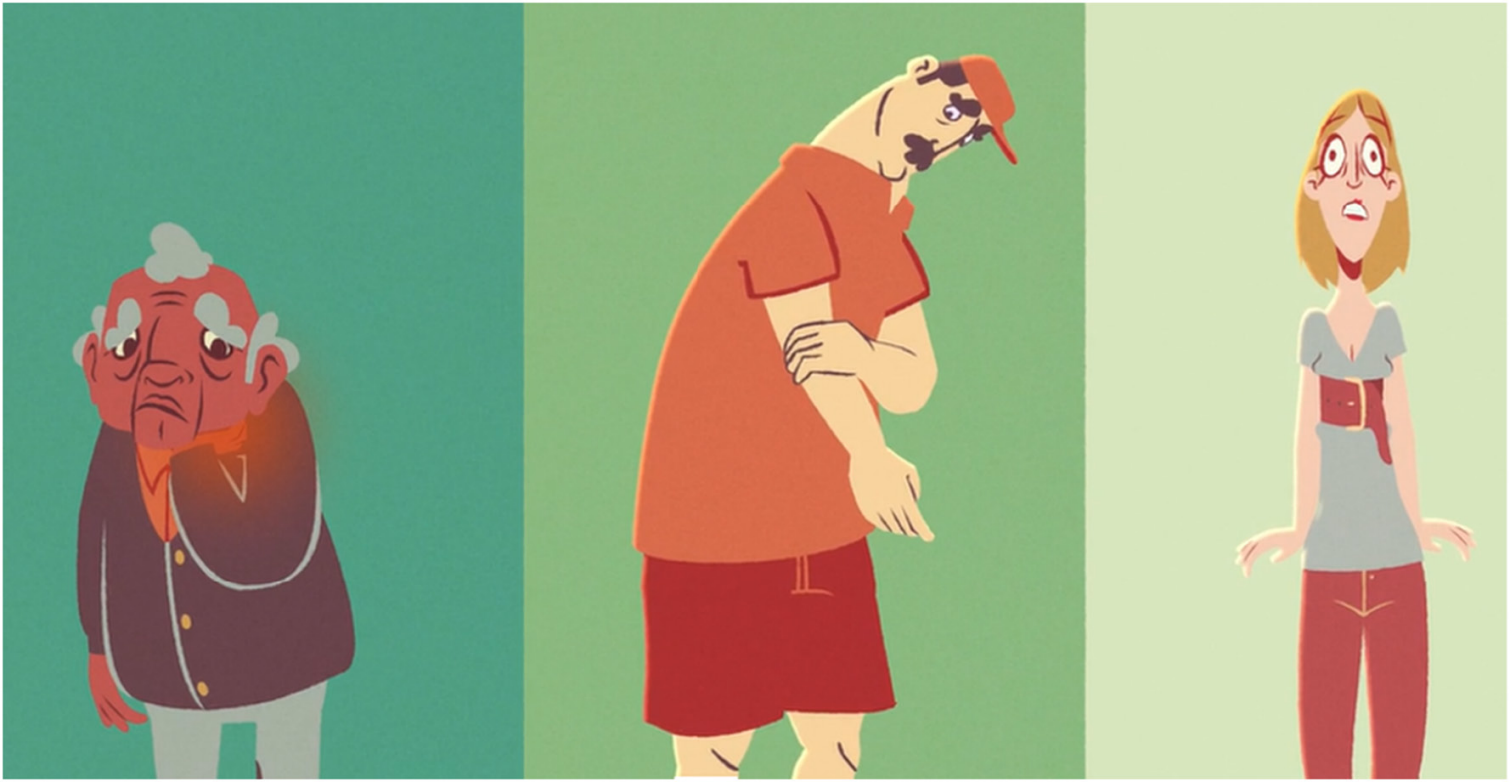
Example screenshot of animation

**FIGURE 2:**
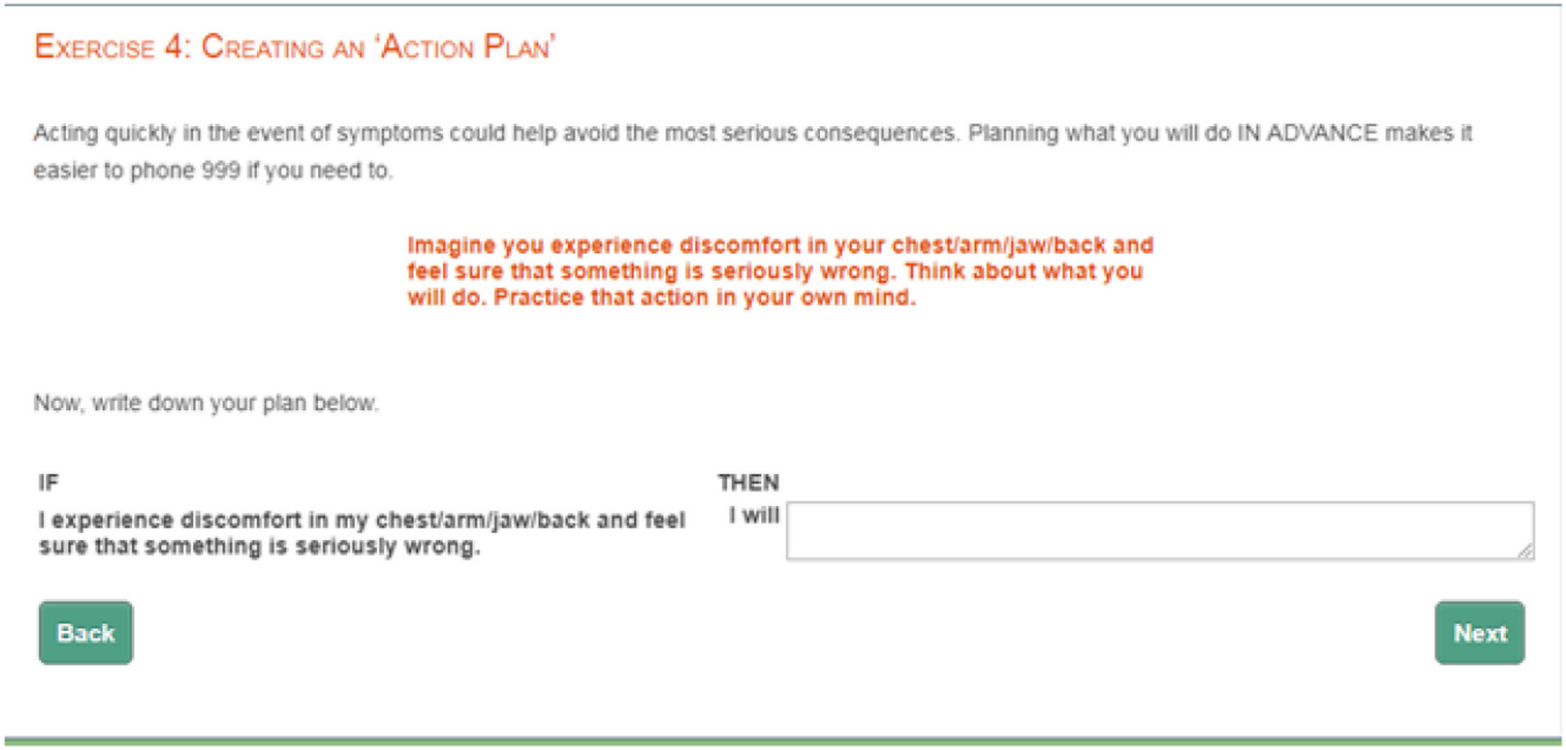
Example screenshot of interactive exercise

Participants in the ‘text+visual’ intervention group received the usual care specified above plus a specifically developed ‘text+visual’ BCT‐based intervention, comprising the 12 BCTs identified from the SR and expert consensus study described earlier (see Box 1). Details of how the intervention was developed are described in the published protocol (Farquharson et al., 2017). The ‘text+visual’ intervention comprised an animated video (just under 8 minutes in length) plus short on‐screen interactive exercises and was hosted online within the IME. Nine BCTs were embedded within the animation, which tells the ‘delay stories’ of 3 different characters (see Figure 1. for an example screenshot) and seven in the interactive exercises (see Figure 2 for an example screenshot) (Four BCTs were used in both the animation and interactive exercises).

#### Usual care plus ‘text‐only’ BCT‐based intervention (intervention group 2)

Participants in the ‘text‐only’ BCT‐based intervention group received the usual care specified above plus a ‘text‐only’ BCT‐based intervention. This was developed in the same way as the ‘text+visual’ BCT‐based intervention but without the visual elements (i.e. animation). Instead, the voiceover from the animation was displayed in text on screen and narrated in audio. The interactive exercises were delivered in the same way as in the ‘text+visual’ BCT‐based intervention.

#### Checks to test BCTs are reliably present within the interventions

Checks were undertaken by two behaviour change experts, external to the project and blind to the BCTs intended for inclusion in the interventions and usual care information. Eleven of the 12 intended BCTs were confirmed as present in both the ‘text‐only’ and ‘text+visual’ versions of the intervention by at least one expert (9 were confirmed present by both experts). The BCT not identified by either coder (BCT 15.2 ‘Mental rehearsal of successful performance’) was made more explicit by adding the following text to the interactive part of the intervention: ‘Please take a few minutes to imagine in your own mind acting this plan out if you have symptoms and feel that something is seriously wrong’.

### Data collection

#### Self‐report questionnaire

Participants were asked to complete a 30‐item questionnaire (see Supplemental material) assessing the following:

##### Socio‐demographic information

Age, ethnic origin, employment status, educational level, marital status and living arrangements. This information was collected before participants were randomized or presented with any scenarios.

##### Primary outcome measures

*Intention*: Informed by the theory of planned behaviour (Ajzen, 1991b), participants' intentions to phone an ambulance immediately was assessed in response to each scenario using a single Likert‐type item: ‘*For these symptoms, after this amount of time, I would phone an ambulance immediately’*, scored 1 = strongly disagree to 7 = strongly agree.

##### Secondary outcome measures

###### Illness and symptom perceptions

The Brief Illness Perception Questionnaire (B‐IPQ) (Broadbent et al., 2006) was used to measure participants' illness representations in relation to the symptoms presented in each scenario. The 9‐item questionnaire measured the five components that make up a person's perception of their illness—identity, cause, timeline, consequences and cure–control (see protocol for more information).

###### Cognitive determinants of intention

Informed by the theory of planned behaviour (TPB) (Ajzen, 1991b), the questionnaire included three items assessing attitudes, subjective norms and perceived behavioural control.

###### Self‐efficacy

Informed by the social cognitive model (Bandura, 1998), people's generic self‐efficacy to call an ambulance immediately was assessed once before and once after the intervention. Participants were asked to rate how certain they are that they could phone an ambulance immediately in nine different situations which varied in how difficult it would be to phone an ambulance (e.g. if you were out with friends).

### Study materials

#### Symptom scenarios

The study used varied symptom scenarios to elicit participants' intentions and cognitions about seeking help. To avoid response bias and memory effects, a randomly generated *range* of symptom scenarios from a pool of 32 were used and presented in a random order before and after the intervention (see protocol for more information about scenarios). Each participant was presented with at least 10 scenarios, 5 before and 5 after the intervention (the first phase of participants received 8 before and 8 after but was reduced on 11 July 2017 when it became apparent it was taking participants longer to complete than anticipated). At least two of the scenarios before and after represented a situation where an ambulance should be called. These are referred to as ‘trigger scenarios’, for example chest or arm discomfort, lasting >15 minutes, either mild or severe, in isolation or with accompanying symptoms.

#### Data collected automatically by the web platform

The following measures were recorded by the web platform:

- The amount of time spent by participants on the web pages containing the usual care information and the ‘text‐only’ or ‘text+visual’ BCT‐based interventions to provide a likely indication of whether or not participants viewed the presented information and how well they engaged with it (Danaher & Seeley, 2009).
- Participants' reaction time when responding to the behavioural intention question upon reading each scenario. This measure of promptness/delay in responding could be considered a proxy for the difficulty of the decision.

### Study procedure

Participants were asked to provide baseline socio‐demographic information, and then, the study task itself involved three phases: before, intervention and after, to be completed within a single study session.

#### Before the intervention

Participants were first asked to complete the generic self‐efficacy questions in relation to phoning an ambulance immediately. The participants were then presented on screen with a randomly selected series of 5 or 8 scenarios (at least 2 trigger scenarios and the remainder non‐trigger). Following each scenario, participants were asked about their intention to phone an ambulance immediately in response to the symptoms described and to complete measures of illness perceptions, attitudes, perceived social norms and perceived behavioural control.

#### Intervention

In the intervention phase, participants received either the ‘text‐only’, ‘text+visual’ or the usual care information only, according to their randomized group allocation.

#### After the intervention

Participants were presented with another randomly selected series of scenarios (different to those at baseline but with the same proportion of trigger scenarios). Following each scenario, they were asked about their intention to call an ambulance immediately in response to those symptoms and to complete the measures of cognitions about those symptoms. To maximize collection of the primary outcome data (i.e. intention) during the post‐intervention phase, participants' intentions were elicited first following each scenario. Following completion of the post‐intervention task, participants were asked to complete the generic self‐efficacy questions in relation to phoning an ambulance immediately.

### Ethics considerations

Ethics approval was provided by Scottish Research Ethics Committee 01, Ref (Norris, 2005)/SS/0155. All potential ethical issues were previously described in the published protocol (Farquharson et al., 2017).

### Data analysis

Two sets of ‘total scores’ were calculated for primary and secondary outcomes for both pre‐ and post‐intervention phases. One set contained the total score for trigger scenarios, derived by taking the mean of scores in response to the scenarios containing the necessary triggers of an emergency ambulance response. The other set contained the total scores for non‐trigger scenarios, derived by taking the mean scores in response to the scenarios containing non‐trigger symptoms. Where data were missing, total scores were derived where at least one score was recorded. Descriptive statistics were used to summarize the primary and secondary outcome measures pre and post‐intervention and change in scores.

To assess the effect of intervention on the primary outcome variable intention to seek help immediately, we used analysis of covariance (ANCOVA) to compare the three study groups using the baseline level of intention as a covariate. Planned comparisons were performed between (i) usual care and ‘text‐only’ BCT‐based intervention (ii) usual care and ‘text+visual’ BCT‐based intervention (iii) ‘text‐only’ and ‘text+visual’ BCT‐based interventions. Similar analyses were performed to test the effect of interventions on the targeted cognitions. Mediation analysis was planned but was not appropriate because neither a main effect of the intervention nor an effect on the mediators was identified. Instead, a multiple regression analysis of intention change scores was conducted to assess how much of the variance in intention was explained by the theoretical predictors.

The same analytical approach (descriptive ‘before and after’ and ANCOVA) was taken to assess for any undesired effects of the intervention on intentions to seek help immediately for scenarios that were not likely ACS.

**FIGURE 3:**
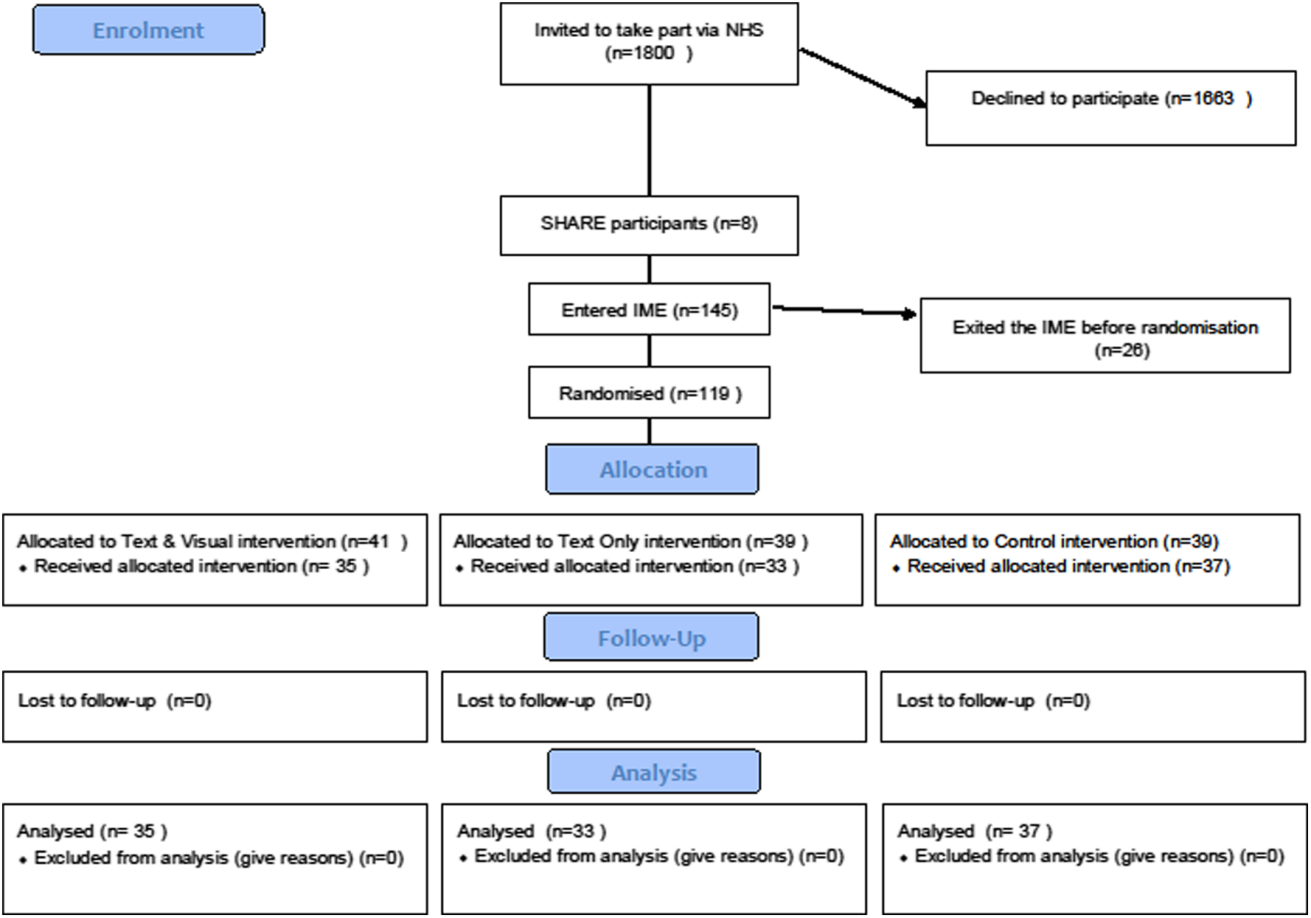
CONSORT diagram

All analyses were by intention‐to‐treat. Item averages were computed where there was at least one non‐missing value. Individuals with missing values were removed from analyses. All data are reported in line with CONSORT guidance for statistical reporting of trial data (Schulz et al., 2010), and a CONSORT style flow chart is provided in Figure 3. (http://www.consort‐statement.org/).

## RESULTS

A total of 1800 patients (*n* = 802 in NHS Board Area 1, *n* = 677 in NHS Board Area 2, *n* = 321 in NHS Board Area 3) were invited to take part via hospital staff between 17 February 2017 and 9 October 2017. Eight participants were identified via SHARE. See CONSORT diagram (Figure 3), below.

In total, *N* = 145 consented to take part and undertook the IME between 11 March 2017 and 9 November 2017. Participation was highest for those invited in person than those invited by letter (32% vs 7%, χ^2^ = 40.5; df = 1; *p* < .001). Overall participation as a percentage of those invited was 7.6% (137/1800), with participation higher at the original study site NHS Board Area 1 (*n* = 88/802; 11%) than at the sites added later: NHS Board 2 (*n* = 38/677; 5.6%) and NHS Board 3 (*n* = 11/321; 3.4%). A total of 119 patients were randomized to allocated intervention (41 to ‘text+visual’, 39 to ‘text‐only’ and 39 to control); the remaining 26 exited the online experiment prior to randomisation. Small numbers of participants in each group exited during the receipt of the intervention; the number who completed the interventions in each group is reported in Figure 3. Primary outcome data were available for *n* = 106; 93 of those completed the IME in full, completing primary outcome data and all subsequent post‐test measures.

Participants were significantly less likely to come from an area of social deprivation than non‐participants (see Table 1, below) and more likely to have had a diagnosis of Unstable Angina rather than the more clinically serious forms of ACS, myocardial infarction (19% vs 8%, *χ* ^*2*^ = 12.3 (*χ* ^*2*^ = 8.8; df = 1*; p* = .003)), but participants and non‐participants did not differ in relation to age, gender or time since ACS event. 68% of the sample were male, average age was 65 years, and median Carstairs quintile (1‐least affluent, 5‐most affluent) was 4. *N* = 53 (39%) had a clinical diagnosis of STEMI, *n* = 73 (53%) NSTEMI and *n* = 11 (8%) unstable angina. There were no significant differences between study arms in relation to any of the studies variables.

**TABLE 1:** Description of participants and non‐participants

	Participants	Non‐participants	Difference (95% CI)	*p*‐value
	Max *n* = 145	Max *n* = 1663		
	Mean (SD)			
Age in years	64.6 (9.6)	65.9 (12.4)		
Number of days from ACS event: Mean (SD)	130.8 (52.8)	125.3 (51.7)	5.42 (‐3.6, 14.5)	.241
Number of days from ACS event: Median (IQR)	142 (88 to 180)	129 (82 to 174)		

	*n* (%)			
Male gender	99 (68.3)	1103 (66.3)		
White ethnic group	145 (100)			
Employed	55 (37.9)			
Educated to degree level or above	53 (36.6)			
Married/ living as married	107 (73.8)			
Lives alone	30 (20.1)			
Rates current health as good or better	95 (65.5)			
Carstairs quintile 2011 mean (SD), *n*	3.53 (1.41)	2.60 (1.52)	0.93 (0.66 to 1.20)	*.000*
Carstairs quintile 2011 (IQR), *n*	4 (2 to 5)	2 (1 to 4)		

Participants spent an average of 52 minutes undertaking the IME. Average time spent on engaging with the usual care information was 2.5 minutes, with text+visual intervention 26 minutes and with text‐only intervention 24 minutes. The breakdown of the average time participants spent engaging with the various elements of the interventions is presented in Table 2.

**TABLE 2:** Time spent on behaviour change techniques in two intervention groups

	Animation/Text‐only	Consequences	Action planning	Problem‐solving	Pros and Cons
		Mean time in minutes (SD)			
Text+visual	7.3 (4.4)	4.1 (5.3)	3.0 (2.6)	5.6 (3.8)	6.8 (7.6)
Text‐only	6.3 (3.2)	4.1 (4.0)	2.5 (1.8)	5.2 (2.9)	5.5 (6.1)

Participants actively engaged with both the interventions (see Table 3): listing an average of 4 and 3 consequences of delay in intervention arms respectively. Participants generated an average of 4 action plans each, at least 89% of which contained the desired behaviour (to dial 999). Over 70% typed to share their plan with a significant other. Participants identified an average 3–4 situations that might lead to delay for them and linked these to an average 6–7 potential solutions per situation. Over 80% identified pros of dialling 999 in event of symptoms and 57% identified the cons of delay.

**TABLE 3:** Engagement with behaviour change‐based interventions

	Text+visual	Text‐only
	*n* = 41	*n* = 35
	Mean (SD)	
Consequences		
Number of consequences of delay identified	4 (2.6)	3 (2.9)
Action planning		
Average number of action plans identified	4 (0.8)	4 (1.0)
Proportion of participants who generated an action plan	88%	94%
% of plans which involved the desired behaviour (dial 999)	89%	93%
% chose to email/print action plan	72%	74%
Problem‐solving		
Number of situations identified that might lead to delay (n/11)	4 (1.6)	3 (1.9)
Average number of solutions identified per situation	7 (3.1)	6 (3.0)
% of identified situations linked with a solution	100%	100%
Pros and Cons		
% identified pros of dialling 999 in event of symptoms	85%	83%
% identified cons of dialling 999 in event of symptoms	55%	54%
% identified pros of delaying	45%	35%
% identified cons of delaying	57%	57%

Aim 1: To determine the effectiveness of the theory‐based interventions (‘text+visual’ and ‘text‐only’ BCT‐based interventions) against usual care in changing patients' intentions to phone ambulance immediately with symptoms of ACS ≥15 minutes duration.

Baseline intentions to seek help immediately with symptoms of ACS (scored 1 low to 7 high) were high with participants scoring an average of 5.5 (SD = 1.6) across trigger scenarios (i.e. those that include symptoms of ACS) and 3.2 (SD = 1.6) for non‐trigger scenarios.

ANCOVA of post‐intervention intention scores for trigger scenarios between study groups with pre‐intervention intention scores as covariate revealed no effect of study group (F = 2.00, *p* = .14) and no effect of pre‐intervention intention (F = 3.06, *p* = .083) (Table 4).

**TABLE 4:** Post‐intervention intention scores: Comparison between intervention groups

Allocation	*N*	Mean	SD	CI_low	CI_high
Control	37	5.74	1.45	5.27	6.21
Text+visual	35	6.34	1.03	6.00	6.68
Text‐only	33	5.76	1.50	5.25	6.28

For trigger scenarios, changes in intention were greater in the text+visual group than usual care (Cohen's d = 0.18, 95% CI [−0.29, 0.65]), but underpowering means we lack precision and did not reach statistical significance. The effect size for ‘text‐only’ compared with usual care was Cohen's d = −0.02, 95% CI [−0.50, 0.46]. Intentions did not change significantly in the ‘text‐only’ group or control, but there was a significant change in the ‘text+visual’ group (Change score = 0.68, SD = 1.48, 95% CI [0.18, 1.17]) (Table 5).

**TABLE 5:** Intention change scores (post‐pre scores): Comparison between intervention groups

Allocation	*N*	Mean	SD	CI_low	CI_high
Control	37	0.32	2.29	−0.42	1.06
Text+visual	35	0.68	1.49	0.18	1.17
Text‐only	33	0.36	1.91	−0.29	1.02

Aim 2: To determine the more effective mode of delivery by comparing the ‘text+visual’ BCT‐based intervention and ‘text‐only’ BCT‐based intervention, with the usual care group.

Average change scores in intentions in the ‘text+visual’ group were almost double those in the ‘text‐only’ group (0.68 vs 0.36) but again underpowered to detect differences between groups, Cohen's d = 0.18, 95% CI [−0.30, 0.67]. No group was significantly different on intention change scores from any other group at *p* = .05.

We explored whether changes in intention were mediated by changes in the target theoretical constructs. There are three conditions for mediation (Baron & Kenny, 1986): (1) the causal variable needs to be associated with the outcome; (2) the causal variable needs to be associated with the mediator; (3) the mediator needs to be associated with the outcome. Out of the three, we only observed associations between the potential mediators (theoretical constructs) and the outcome (intention). Conditions 1 and 2 were not met: Neither intervention produced a significantly different outcome from control, nor any significant changes in any of the potential mediators when controlling for pre‐intervention scores (Table S1). Condition 3 was met for some mediators with changes in intentions for trigger scenarios significantly correlated (Table S2) with a change in illness representations (increased illness concern, more serious perceived consequences, stronger emotional response, increased timeline, more positive perceptions of treatment control and increased coherence [understanding] of the symptoms); more positive attitudes; and more positive perceived social norms to phoning an ambulance. There was no significant correlation between participants' changes in intention for trigger scenarios and self‐efficacy or perceived behavioural control. In conclusion, mediation analysis was deemed inappropriate due to conditions not having been met.

With regard to significant changes before and after the intervention, longer expected duration of symptoms (timeline) was seen in the ‘text+visual’ group and greater personal control in the ‘text‐only’ group. No significant changes in theoretical constructs relating to trigger scenarios were seen in the usual care group (see Tables S3 and S4).

Aim 3: To investigate any unintended consequences of the intervention on intentions to phone an ambulance for non‐life‐threatening symptoms.

Changes in intention for non‐trigger scenarios did not differ significantly between the 3 groups (underpowered to detect) (Table S5). Intentions to seek help immediately with *toe discomfort or dysuria of > 15 mins duration* were not changed significantly in either the ‘text+visual’, ‘text‐only’ or control groups (see Table S6). Intentions to seek help immediately for non‐urgent symptoms were not significantly different between the ‘text‐only’ and the ‘text+visual’ group.

### Time to respond to items as a proxy for ease of decision

There was no difference in time to respond to intentions for scenarios between allocation groups or trigger and non‐triggers scenarios. There was no correlation between response times and responses to the Intention measure.

## DISCUSSION

We have systematically developed and carefully tested two versions of a BCT‐based intervention to reduce patient delay with ACS. The trial design which required participants to indicate what they would do in relation to varied symptoms scenarios and comparing their responses to pre‐specified ACS scenarios before and after intervention was a rigorous test of the intervention. Our within‐group results have shown that a BCT‐based ‘text+visual’ intervention can significantly increase intentions to seek help immediately with symptom of ACS, even in the context of high baseline intention and confidence about phoning an ambulance for symptoms. Furthermore, we have shown that this increase in intentions can be achieved without unintended changes in help‐seeking for non‐urgent symptoms.

Importantly, the intervention appears to have been successful in achieving the desired changes in the relevant theoretical constructs—almost all change scores were in the direction that theory would suggest were likely to increase intentions (i.e. positive changes), statistically significant changes after the intervention being seen in perceptions of timeline in the ‘text+visual’ group and in personal control in the ‘text‐only’ group. No significant changes in theoretical constructs relating to trigger scenarios were seen in the usual care group.

### Mechanism of action

A particular strength of the study is the data collected regarding potential theoretical mechanisms of action which means we have a good understanding of ‘how’ the intervention works which will usefully inform further refinement of this intervention, as well as the development of others (Sumner et al., 2018).

#### Results consistent with behavioural theories

Change in intention was associated with changes in people's illness representations about symptoms, creating more positive attitudes about phoning an ambulance in this situation and creating the perception that others would approve. The changes in illness representations associated with change in intention (increased illness concern, more serious perceived consequences, stronger emotional response, increased timeline, more positive perceptions of treatment control and increased coherence) are all in the direction predicted by the underlying theory (Broadbent et al., 2015; Leventhal et al., 1984) and consistent with previous research which has shown perceptions of more severe consequences to be associated with shorter delay in ACS (Farquharson et al., 2012; Walsh et al., 2004). Bandura (1998), Farquharson et al. (2012) found a stronger emotional response to be associated with shorter delay in patients with possible ACS, and Jensen et al. (2016) found increased perceptions of treatment control associated with shorter delay in people with symptoms of colorectal cancer. Increased coherence has previously been negatively correlated with patient‐related delay amongst people with rheumatoid arthritis (Van der Elst et al., 2016). More positive attitudes and increased perceived social norms being associated with increased intention is in line with the predictions of the theory of planned behaviour, adding to the significant evidence base around this theory (Ajzen, 1991b; McEachan et al., 2011).

#### Why were perceived behavioural control and self‐efficacy not associated with intention in this study?

Comprehensive meta‐analysis of previous research has confirmed perceived behavioural control as a strong predictor of intention across a number of behaviours (McEachan et al., 2011), but although confidence about phoning an ambulance (the similar constructs of Self‐efficacy or perceived behavioural control) increased in the intervention groups, changes in these constructs were not significantly associated with increased intentions in our study. There are a number of possible explanations why this was—it may be there is a ceiling effect and that our participants (who had high baselines levels of self‐efficacy and perceived behavioural control) already had sufficient confidence and that further increases beyond this level have little additional effect. It might be that we did not achieve great enough increases in confidence to translate into changes in intentions. The author of the TPB does suggest that the relative importance of attitude, subjective norm and perceived behavioural control in the prediction of intention is not expected to be the same across all behaviours and situations, and it may be that perceived behavioural control is less critical in relation to the behaviour of phoning an ambulance with symptoms of ACS (Ajzen, 1991b). However, given that perceived behavioural control is a strong predictor of intention in general (mean ρ = 0.54 (McEachan et al., 2011)), taking additional steps to increase perceived behavioural control seems warranted. We will refine the intervention by adding further BCTs to increase self‐efficacy/perceived behavioural control about phoning an ambulance with symptoms of ACS. The Delphi study suggested 15.1 Verbal persuasion about capability, 15.2 Mental rehearsal of successful performance and 15.4 Self‐talk would be useful in this regard, techniques that the ‘Theory and Techniques’ Online Tool confirms as having ‘strong links’ to the mode of action of increasing beliefs about capabilities (Carey et al., 2019; Johnston et al., 2020).

#### BCTs as active ingredients

The lack of change in theoretical constructs in the usual care group in contrast to the intervention groups provides good evidence that BCTs are acting as ‘active ingredients’ and influencing cognitions in the direction suggested by theory. Careful attention to BCT content in future interventions is recommended.

The RAPiD intervention could be of significant utility to patients discharged from hospital after ACS (over 100,000 every year in the United Kingdom (Foundation, B.H, 2022)) and could also have wider application as a public health intervention if effective long‐term. The digital nature of the intervention means that complex information for patients about what to do in the event of symptoms with perfectly preserved fidelity can be made available to patients beyond hospital discharge (when the advice is most needed).

Unfortunately, we were unable to achieve our desired sample size, a problem which was further compounded by challenges with missing data and ceiling effects. Thus, we were underpowered to detect statistically significant differences between groups. However, results are in the anticipated direction, suggesting a positive effect of the BCT‐based intervention, particularly when delivered visually and engagement with the interventions was very high. Given the significant advantages this medium holds in terms of ease of access, suitability for those with low literacy preserved fidelity, and yet without requirement for health care professional input, we believe the intervention shows significant promise and is worthy of further testing.

### Limitations

The study has been conducted as per a pre‐published protocol and with a high level of rigour, in a representative clinical sample; however, inevitably there are some limitations. The proportion of participants invited into this online experiment who took part was low, and they may not be representative of the overall population. We reduced the number of scenarios during the experiment which was not ideal but necessary when it became clear participation was more time‐consuming than expected, threatening completion of the task and the validity of responses. We do not believe this will have affected results because all participants still received a mixture of ACS and non‐ACS scenarios, and results show they distinguished between them. We acknowledge that time spent on the online platform is a crude measure of engagement with the intervention and further exploration of participants' experiences of the intervention would be of value. We also recognize that what people say they will do in response to hypothetical situations may not translate into the desired behaviour in the event of real‐life experience of symptoms. Nonetheless, the pattern of responses from participants shows they had clearly differentiated responses to trigger and non‐trigger scenarios providing confidence that participants were engaging meaningfully with the decisions they were asked to make. Establishing whether the interventions are effective in modifying patient behaviour in the event of actual symptoms will be the next step in our research. The study was underpowered to be precise about differences between groups, and so we cannot be conclusive about intervention effectiveness. However, the fact that cognitions changed in the direction predicted by theory in the intervention groups and that a significant change in intention was observed in the context of high baseline intentions suggests a positive effect.

We recommend future research pursue a theory‐based, visual approach and move beyond responses to hypothetical scenarios by measuring behaviour with actual ACS symptoms.

## CONCLUSION

A robust randomized controlled IME of a systematically developed BCT‐based intervention suggests that a ‘text+visual’ BCT‐based intervention may significantly increase intention to phone an ambulance with symptoms of ACS without unwanted effects on intentions to seek help for other (non‐urgent) symptoms. The intervention is engaging, has high fidelity and primarily appears to achieve its effect by changing illness representations about symptoms of ACS, creating more positive attitudes about phoning an ambulance in this situation and creating the perception that others would approve. These data provide an excellent basis for informed refinement of the intervention and further testing of the effect of the intervention on actual behaviour is warranted.

## AUTHOR CONTRIBUTIONS

**Brian WILLIAMS:** Conceptualization; funding acquisition; methodology; writing – review and editing. **Barbara Farquharson:** Conceptualization; formal analysis; funding acquisition; methodology; project administration; supervision; writing – original draft; writing – review and editing. **Claire JONES:** Funding acquisition; methodology; software; writing – review and editing. **Jan SAVINC:** Formal analysis; writing – review and editing. **Karen SMITH:** Conceptualization; funding acquisition; methodology; writing – review and editing. **Marie JOHNSTON:** Conceptualization; funding acquisition; methodology; supervision; writing – review and editing. **Mark GRINDLE:** Resources; writing – review and editing. **Nadine DOUGALL:** Formal analysis; funding acquisition; methodology; writing – review and editing. **Purva ABYHANKAR:** Project administration; writing – original draft; writing – review and editing. **Shaun TREWEEK:** Funding acquisition; methodology; writing – review and editing. **Stephan DOMBROWSKI:** Funding acquisition; methodology; writing – review and editing.

## FUNDING INFORMATION

This work was supported by the Chief Scientist Office, Scotland: Grant number CZH/4/1025.

## CONFLICT OF INTEREST

All authors declare no conflict of interest.

## Supporting information


Table S1

Table S2

Table S3

Table S4

Table S5

Table S6

Supplemental Material

## Data Availability

The data that support the findings of this study are available from the corresponding author upon reasonable request.
