# Supplementary material for: Reducing patient delay in acute coronary syndrome: Randomized controlled trial testing effect of behaviour change intervention on intentions to seek help
Source: Br J Health Psychol. 2022 Aug 8;28(1):188–207. doi: 10.1111/bjhp.12619 (PMC10086951; doi:10.1111/bjhp.12619)
Supplement: Supplementary file 1 — Table S1 [file BJHP-28-188-s006.docx]

Supplemental file 1: ANCOVA results for all measured variables. In all cases, the post-intervention scores were entered as the dependent variable, with intervention as the independent variable and pre-intervention score as covariate. For intervention DF=2, and for pre-intervention scores DF=1. MSE is Mean Squared Error, and is calculated by dividing the residual sum of squares by the residual DF. < .01; * < .05; ** < .01; *** < .001

| Construct | Intervention | Pre-intervention score |  |
| --- | --- | --- | --- |

|  | F | p | F | p | MSE |
| --- | --- | --- | --- | --- | --- |
| Intention | 2.00 | 0.14 | 3.06 | .083 . | 1.77 |
| SelfEfficacy | 0.29 | 0.75 | 73.20 | <.001 *** | 160 |
| Attitude | 1.73 | 0.18 | 0.90 | 0.35 | 1.85 |
| PSN | 1.55 | 0.22 | 8.05 | .0057 ** | 1.64 |
| PBC | 0.16 | 0.85 | 37.90 | <.001 *** | 2.15 |
| BIPQ.COMPOSITE | 1.45 | 0.24 | 50.50 | <.001 *** | 171 |
| BIPQ.Concern | 1.78 | 0.18 | 43.50 | <.001 *** | 2.99 |
| BIPQ.Consequences | 0.96 | 0.39 | 20.00 | <.001 *** | 4.53 |
| BIPQ.Emotion | 0.13 | 0.88 | 39.50 | <.001 *** | 4.34 |
| BIPQ.PersonalControl | 1.78 | 0.18 | 20.60 | <.001 *** | 6.75 |
| BIPQ.Timeline | 2.81 | .065 . | 50.10 | <.001 *** | 3.14 |
| BIPQ.TreatmentControl | 1.20 | 0.31 | 52.00 | <.001 *** | 2.67 |
| BIPQ.Understanding | 0.67 | 0.51 | 20.20 | <.001 *** | 4.23 |
