# Supplementary material for: Reducing patient delay in acute coronary syndrome: Randomized controlled trial testing effect of behaviour change intervention on intentions to seek help
Source: Br J Health Psychol. 2022 Aug 8;28(1):188–207. doi: 10.1111/bjhp.12619 (PMC10086951; doi:10.1111/bjhp.12619)
Supplement: Supplementary file 2 — Table S2 [file BJHP-28-188-s004.docx]

Supplemental file 2: Spearman’s rank correlations of intention change scores with other constructs. . < .01; * < .05; ** < .01; *** < .001

| Construct | $rho$ | p.value | n.obs |
| --- | --- | --- | --- |
| Age | -.11 | .28 | 105 |
| Education | .23 | .016 * | 105 |
| Deprivation score | .07 | .5 | 93 |
| Attitude | .61 | <.001 *** | 87 |
| BIPQ.Concern | .61 | <.001 *** | 79 |
| BIPQ.Consequences | .49 | <.001 *** | 92 |
| BIPQ.Emotion | .57 | <.001 *** | 77 |
| BIPQ.PersonalControl | -.03 | .79 | 92 |
| BIPQ.Timeline | .35 | <.001 *** | 92 |
| BIPQ.TreatmentControl | .55 | <.001 *** | 81 |
| BIPQ.Understanding | .41 | <.001 *** | 77 |
| PBC_Trigger_ChangeScore | .14 | .3 | 59 |
| PSN_Trigger_ChangeScore | .57 | <.001 *** | 86 |
| SelfEfficacy_ChangeScore | -.04 | .72 | 99 |
