# Supplementary material for: Reducing patient delay in acute coronary syndrome: Randomized controlled trial testing effect of behaviour change intervention on intentions to seek help
Source: Br J Health Psychol. 2022 Aug 8;28(1):188–207. doi: 10.1111/bjhp.12619 (PMC10086951; doi:10.1111/bjhp.12619)
Supplement: Supplementary file 3 — Table S3 [file BJHP-28-188-s001.docx]

Supplemental file 3:  **Changes in theoretical variables pre & post intervention**
