# Supplementary material for: Reducing patient delay in acute coronary syndrome: Randomized controlled trial testing effect of behaviour change intervention on intentions to seek help
Source: Br J Health Psychol. 2022 Aug 8;28(1):188–207. doi: 10.1111/bjhp.12619 (PMC10086951; doi:10.1111/bjhp.12619)
Supplement: Supplementary file 4 — Table S4 [file BJHP-28-188-s005.docx]

Supplemental file 4: **Change in self-efficacy pre & post intervention**

| **Text&visual** | | | | | |
| --- | --- | --- | --- | --- | --- |
| SelfEfficacy | Pre | Post | ChangeScore | t-test (paired) | Student: equal variances |
| N | 41 | 34 | 34 | t | 1.37 |
| M (SD) | 79.8 (19.4) | 84 (18.6) | 3.28 (13.9) | df | 33.00 |
| CI | [73.8 85.7] | [77.8 90.2] | [-1.4 7.95] | p | 0.18 |
| **‘text-only’** | | | | | |
| SelfEfficacy | Pre | Post | ChangeScore | t-test (paired) | Student: equal variances |
| N | 37 | 31 | 31 | t | 1.66 |
| M (SD) | 77.5 (19.6) | 82.5 (14.8) | 5.41 (18.2) | df | 30.00 |
| CI | [71.2 83.8] | [77.3 87.7] | [-0.987 11.8] | p | 0.11 |
| **Usual Care** | | | | | |
| SelfEfficacy | Pre | Post | ChangeScore | t-test (paired) | Student: equal variances |
| N | 37 | 34 | 34 | t | 0.08 |
| M (SD) | 84.2 (16.9) | 84.5 (16.5) | 0.156 (11.6) | df | 33.00 |
| CI | [78.7 89.6] | [78.9 90] | [-3.73 4.04] | p | 0.94 |
