# Supplementary material for: Reducing patient delay in acute coronary syndrome: Randomized controlled trial testing effect of behaviour change intervention on intentions to seek help
Source: Br J Health Psychol. 2022 Aug 8;28(1):188–207. doi: 10.1111/bjhp.12619 (PMC10086951; doi:10.1111/bjhp.12619)
Supplement: Supplementary file 5 — Table S5 [file BJHP-28-188-s007.docx]

**Supplemental file 5: CHANGE IN INTENTION FOR NON-TRIGGER SCENARIOS: COMPARISON BETWEEN INTERVENTION GROUPS**
