# Supplementary material for: Reducing patient delay in acute coronary syndrome: Randomized controlled trial testing effect of behaviour change intervention on intentions to seek help
Source: Br J Health Psychol. 2022 Aug 8;28(1):188–207. doi: 10.1111/bjhp.12619 (PMC10086951; doi:10.1111/bjhp.12619)
Supplement: Supplementary file 7 — Supplemental Material [file BJHP-28-188-s002.docx]

**Supplemental material**

**Trial of an intervention to guide people in seeking help for specific symptoms**

**Questionnaire**

*(In the order of assessment)*

***The following will be assessed only once, before the intervention***

**About you**

Please answer the following questions about you, all replies will be confidential.

Are you male or female?

Male ☐ Female ☐

How old are you?

**____________________**

How would you describe your ethnic origin? *(please tick one)*

White ☐ Indian ☐ Pakistani ☐ Bangladeshi ☐

Chinese ☐ Black-Caribbean ☐ Black-African ☐ Black-other ☐

Other ☐ (please specify____________________)

What is your current employment status? *(please tick one)*

Full time ☐ Part time ☐ Unemployed ☐

Self Employed ☐ Retired ☐ Other ☐ (please specify____________________)

What is the highest level of education you have received? *(please tick the highest option)*

No formal education ☐ Secondary school education ☐ University Degree ☐

Vocational/Technical qualifications ☐ Other ☐ Please specify____

What is your current marital status? *(please tick one)*

Single ☐ Married/living as married ☐ Divorced/Separated ☐

Widowed ☐ Other ☐ Please specify____

Who do you live with?

I live alone ☐ With spouse/partner ☐ With other family ☐

Sheltered/supported accommodation ☐ Other ☐ Please specify___________

How wold your rate your current health?

Poor ☐ Fair ☐ Good ☐ Very good ☐ Excellent ☐

**The following will be assessed only once before and once after the intervention**

**(Self-efficacy)**

A number of situations are described below that can make it more or less difficult to phone an ambulance. Please rate how certain you are that you **could** phone an ambulance immediately in each situation.

Rate your degree of confidence by moving the bar on the scale from 0 to 100.

|  | Not at all Moderately Highly  certain (0) certain certain (100) |
| --- | --- |
| *If someone told you it was the right thing to do* |  |
| *If you had other important things going on* |  |
| *If you were out with friends* |  |
| *If no one else was present* |  |
| *If a phone was hard to reach* |  |
| *If you had to get help from someone else to phone* |  |
| *If you were not sure what was happening* |  |
| *If you were anxious about the symptoms* |  |
| *If the symptoms occurred at night* |  |
| *If the symptoms occurred on weekend* |  |
| *If you were worried about doing the wrong thing* |  |

***(The following will be assessed in response to each scenario)***

**Few Example Scenarios**

Scenario 1: You experience a mild discomfort in your toe for 2 hours

Scenario 2: You experience a mild discomfort in your arm for 20 minutes. You also feel sick

Scenario 3: You experience a severe discomfort when passing urine for 1 day. You also feel sick

Scenario 4: You experience a severe discomfort in your chest for 5 minutes.

**(Intention)**

| Strongly Disagree | 1 | 2 | 3 | 4 | 5 | 6 | 7 | Strongly agree |
| --- | --- | --- | --- | --- | --- | --- | --- | --- |

**For these symptoms, after this amount of time, I would phone an ambulance immediately**

**(Brief Illness Perception Questionnaire)**

We would like to ask you questions about ***what these symptoms mean to you*** For the following questions, please circle the number that best corresponds to your views:

| **How much would these symptoms affect your life?**  0 1 2 3 4 5 6 7 8 9 10  would not affect would severely  at all affect my life |
| --- |
| **How long do you think these symptoms would continue?**  0 1 2 3 4 5 6 7 8 9 10  a very forever  short time |
| **How much control do you feel you would have over these symptoms?**  0 1 2 3 4 5 6 7 8 9 10  absolutely extreme amount  no control of control |
| **How much do you think treatment would help with these symptoms?**  0 1 2 3 4 5 6 7 8 9 10  not at all extremely  helpful |
| **How concerned would you be about these symptoms?**  0 1 2 3 4 5 6 7 8 9 10  not at all extremely concerned concerned |
| **How well do you feel you would understand these symptoms?**  0 1 2 3 4 5 6 7 8 9 10  wouldn't understand would understand  at all very clearly |
| **How much would these symptoms affect you emotionally? (e.g. do they make you angry, scared, upset or depressed?)**  0 1 2 3 4 5 6 7 8 9 10  Would not would affect me  affect me at all extremely |
| **Please list in rank-order the three most important factors that you believe would have caused** **these symptoms.** ***The most important causes for me:-***   1. **__________________________________** 2. **__________________________________** 3. **__________________________________** |
| **What medical condition do you think these symptoms indicate?**    _____________________________ |

**The following questions are about your views on phoning an ambulance (999) immediately in this situation** *(please circle one number for each statement).*

**(Attitude)**

To phone an ambulance (999) immediately in this situation would be

Useless 1 2 3 4 5 6 7 Useful

Foolish 1 2 3 4 5 6 7 Wise

Inappropriate 1 2 3 4 5 6 7 Appropriate

**(Perceived social norm)**

| People who are important to me would think I  Should not 1 2 3 4 5 6 7 Should  immediately phone an ambulance (999) in this situation |
| --- |
| People who are important to me would  Disapprove 1 2 3 4 5 6 7 Approve  of me immediately phoning an ambulance (999) in this situation |
| Most people in this situation would immediately phone an ambulance (999)  Strongly disagree 1 2 3 4 5 6 7 Strongly agree |

**(Perceived Behavioural Control)**

| Phoning an ambulance (999) immediately in this situation is beyond my control  Strongly disagree 1 2 3 4 5 6 7 Strongly agree |
| --- |
| Phoning an ambulance (999) immediately in this situation is entirely up to me  Strongly disagree 1 2 3 4 5 6 7 Strongly agree |
| It would be difficult for me to immediately phone ambulance (999) in this situation  Strongly disagree 1 2 3 4 5 6 7 Strongly agree |
